# Role of Biomarkers as Predictors of Infection and Death in Neutropenic Febrile Patients after Hematopoietic Stem Cell Transplantation

**DOI:** 10.4084/MJHID.2015.059

**Published:** 2015-10-15

**Authors:** Karin Massaro, Silvia Figueiredo Costa

**Affiliations:** 1 Department of Infectious Diseases, Faculty of Medicine, University of Sao Paulo, Sao Paulo, Brazil; 2 Laboratory of Bacteriology-LIM54, Hospital das Clinicas, University of Sao Paulo, Sao Paulo, Brazil

## Abstract

An ideal marker in the neutropenic population after HSCT is the one which positivetes at the onset of fever, or at most up to 24 hours after its onset, the patients at potential risk for infection due to bacterial and fungi and mortality. Several biomarkers have been used in HSCT patients in the last decade. However, it seems that C-RP and Il-6 are the most useful markers to early detected infection and risk for death

## Introduction

The number of hematopoietic stem cell transplantations has increased significantly over the past decade. The morbidity and mortality due to infectious complications are the major clinical issue in HSCT recipients.^1^,^2^ However, the identification of these infectious complications is still based on clinical criteria, though, mainly in the occurrence of fever.

In patients after HSCT, it’s hard to set infection apart from other causes of fever, such as acute Graft versus host diseases (GVHD), or veno-occlusive disease (VOD) and sinusoidal occlusion syndrome.^3^ The detection of these major transplantation-related complications is essential for the early and proper introduction of antibiotics, immunomodulators or hemostatic treatment. The diagnosis and the evolution of infectious complications may be optimized with the help of early sensitive and specific markers of bacterial and fungal infections. An ideal marker should preferably either precede significant microbiological findings or justify an additional intensive search for an infection focus even in afebrile.^4^ The early prediction of serious bacteremia helps to identify those patients who are more likely to benefit from a combination of drugs and reduce the unnecessary toxicity of additional treatments when there is no need. At the same time, it helps the identification of patients at low risk of complications, which may be treated at outpatient units.^5^,^6^

## Main Biomarkers

### IL-6

Interleukin-6 is a pro-inflammatory cytokine produced by several types of cells, including monocytes, macrophages and endothelial cells. The main form of IL-6 in human plasma consists of a biologically active 45 kDa molecule.^7^

Interleukin-6 (also called hepatocyte stimulating factor), as previously mentioned, is involved with the production of proteins of the acute phase, such as C-reactive protein (CRP).^8^ The production and secretion of IL-6 may be induced by a great variety of stimuli, including infection by Gram-positive and Gram-negative bacteria, viruses, lipopolysaccharides, TNF-α, interleukin 1β, gamma interferon and platelet-derived growth factor.^9^–^11^ The kinetics of this cytokine is very fast (induction in less than 1–2 hours), but concentrations may decline within very short time.

IL-6 does not react specifically to an infectious stimulus (it increases both in viral infections and bacterial ones, and in other situations, such as autoimmune disease and tissue trauma) and presents considerable fluctuations in daily levels, suggesting temporary activation or suppression of the immune reaction.^12^

### C-reactive protein

The CRP is an acute phase protein, whose serum concentration is remarkably increased right after the occurrence of aggression to the body. Its plasma concentrations are usually below 10 mg/L.^13^ It is formed by a complex constituted by five polypeptide subunits synthesized by the liver, not covalently bonded, with an approximate molecular weight between 115 kDa and 140 kDa.

The main stimulus mediator to the production of CRP is IL-6; however, other cytokines, such as IL-1 and TNF-α are also involved in the process. 14–15 Since IL-6 is the main response mediator of the acute phase, many clinical conditions, besides inflammation, may cause a rise in the CRP. Additionally, CRP has the disadvantage of not raising above its reference top level, prior to 8–12 hours after the onset of the inflammatory process.^16^

### Procalcitonin

Procalcitonin (PCT) is a 14 kDa protein, coded by gene Calc-1, together with calcitonin and katacalcin (KC). It consists of 114 to 116 amino acids. It is usually found in the C cells of the thyroid, as a pre-hormone of calcitonin. However, in 1993, it was found that serious bacterial infections caused a dramatic release of PCT in the extracellular space. The concentrations of the soluble protein in the serum or plasma, in the normal population, are usually below 0.1 ng/mL (normal range). In case of levels above 0.5 ng/mL, the diagnosis of sepsis must be considered.^17^ In a study in which we provided dosages of PCT and CRP in 52 inpatients, with hemopathies and febrile neutropenia, the PCT average was significantly higher in cases with serious infection (6.7 ng/mL versus 0.6 ng/mL).^18^ By using a cut-off value of PCT, above 0.245 ng/mL, we observed a 100% of sensitivity and 69.2% of specificity in serious infection, suggesting the use of PCT as a diagnostic marker for severe systemic infection in this population, to the detriment of CRP. This cut-off value was lower than that of the study by Giamarellou et al. (0.5 ng/mL) in 2004.^19^

The pharmacokinetic properties of PCT enable its early use (within six hours), as well as its follow-up (from 24 hours on) as infection and inflammation marker. Another interesting characteristic of this molecule is that its half-life is independent of the renal function.^20^

### IL-8

IL-8 is known as a pro-inflammatory cytokine, which is involved in local and systemic inflammatory reactions. Short and high peaks were observed a few days after VOD.^21^ IL-8 can be released by endothelial cells activated in response to TNF-α and it is a potent activator of neutrophils, which may contribute to tissue damage.^22^

## Studies of Biomarkers of Infection in Febrile Neutropenia after HSCT

PCT, IL-6 and CRP cannot be utilized to differentiate Systemic Inflammatory Response Syndrome (SIRS) from infection, in patients who receive ATG or similar within up to three days after the application of the drug. PCT, CRP and IL-6 have limited value in the diagnosis of infection during the administration of ATG or other anti T-cell antibodies, such as OKT-3.^23^ On the other hand, Blijlevens et al. (2000)^24^ found increased levels of PCT in isolated cases of GVHD.

### Procalcitonin

There can be a slight induction of PCT after HSCT, as when it occurs in chemotherapy, with levels rarely above 0.5 to 1 ng/mL. That was observed, for instance, in children with ALL, AML, NHL with B and LH cells.^25^ In cases of serious infections or sepsis, these patients may also induce high levels of PCT. If neutropenia is severe, the induction of PCT is reduced; however, the synthesis is not totally suppressed.^26^–^27^ Some considerations must be made to justify the different results in two of the studies mentioned. In the study by Blijlevens et al. (2000),^24^ the limited number of patients harmed the statistical analysis of the results. Zintl et al. (1996) 28 showed, in a study with patients submitted to HSCT with prior myeloablative chemotherapy, that PCT increases in systemic bacterial infections and is strictly related to the septic shock and also that higher levels indicate bad prognosis. A study that evaluated PCT in neutropenic hematological patients with mucositis revealed that it represents an important tool to predict the occurrence of major infections in patients with febrile neutropenia, even in the presence of associated complicating conditions, such as mucositis and GVHD.^29^ A lower threshold value (e.g. PCT > 0.25 ng/mL) increases the diagnostic reliability in case of a serious infection, even in patients with neutropenia.^30^–^31^ This reduced increase may happen since the adhering monocytes, among other factors, are necessary for the induction of PCT. Even if the immune response is overall weakened in neutropenic patients, and also by the use of corticosteroids or cyclosporine for the treatment of autoimmune diseases or after transplantation, the induction of PCT is not totally suppressed.^32^ In case of neutropenia, the severity of the systemic inflammatory response cannot often be evaluated, since the “fever” symptom is weakly associated with the gravity of the sepsis or prognosis of the disease. In an international multicentric study, 158 patients with neutropenia and fever were evaluated.^19^ A cut-off value of 5 ng/mL determined a sensitivity and specificity for the diagnosis of severe sepsis of 83% and 100% respectively. In concentrations below 0.5 ng/mL, the severe infection would be unlikely. In case of fungal infections, the levels of PCT were 1.17 ± 0.44 ng/mL on the first day of diagnosis, with a quick downward trend. Twelve patients died. On the first day of fever, they had levels of PCT of 20.45 ± 4.48 ng/mL (average, standard deviation). Similar data were reported in other studies. 24,26,33 A study that compared the value of PCT, neopterin, CRP, IL-6 and IL-8, as diagnostic markers for such purpose, showed an area under the ROC curve in the distinction of bacteremia by Gram-negative from all the other causes of fever of 0.863 (IC 95% 0.74–0.98) and results not significant for the other markers. 34

Ortega et al. (2004)^35^ showed PCT levels in patients after HSCT with FUO of 0.3 ± 0.2 ng/mL (average, standard deviation), and with infections microbiologically documented in the range of 0.5 ± 0.7 ng/mL. In cases of extended fever (over five days) and on suspicion of aspergillosis, the levels of PCT were even higher (10.1 ± 6.7 ng/mL).

In a recent study that evaluated the relevance of ultrasensitive CRP and PCT levels in patients after HSCT, it was verified that the ultrasensitive CRP was higher in the patients with aGVHD and also in the cases of sepsis. The increased levels of PCT were associated only with a bacterial infection. Only the levels of PCT could be used to differentiate infection from other complications related to transplantation.^36^ In the study mentioned, PCT, besides not increasing in GVHD, did not increase in fungal infections, although the bias of that study was the size of the sample (n = 35).

### C-reactive protein

A study with adult patients submitted to allogeneic HSCT (n = 137) showed that the pre transplantation CRP and serum ferritin levels can be predictors of bacterial complications.^37^ This study has the limitations of being retrospective, without standardization of different hematological diseases and limited number of samples. Likewise, Pihusch et al. (2006)^38^ found high CRP levels both in infections after HSCT and in aGVHD cases. In this study, which analyzed the levels of PCT, CRP, and IL-6, prospectively, in 350 patients after HSCT, conditioning of some patients included anti-thymocyte globulin (ATG). It is well known that ATG, which is a pool of heterologous antibodies against T-cells of direct form (opsonization and lysis via complement activation), causes early increase of TNF-α, CRP, IL-6 and PCT (few minutes after administration).^23^,^39^

### Interleukin-6 (IL-6)

Some studies show that IL-6 is a sensitive marker for acute infections in febrile neutropenia and it can be used as a marker of unfavorable results in this situation.^40^–^42^ The values of IL-6 (measured by the Enzyme-linked immunosorbent assay [ELISA] method) for febrile neutropenia vary in several studies. In the study by Steinmetz et al. (1995),^8^ the level of detection threshold was of 3 pg/mL, with intratest variations of < 15% (in the 3–100 pg/mL range) and < 20% for values > 100 pg/mL. A significant increase of IL-6 was considered as an increase higher than 20 pg/mL or 30% for levels < 100 pg/mL and ≥50 pg/mL or 20% for levels > 100 pg/mL. A cut-off value of IL-6 of 297 pg/mL presented sensitiveness of 62%, positive predictive value (PPV) of 50% and negative predictive value (NPV) of 70% (p = 0.016) for the identification of infection in neutropenic patients.^33^

### Interleukin-8 (IL-8)

The rapid increase of IL-6 and IL-8, quickly detectable on the first days after the HSCT, may either be the mere reflection of the severity of the inflammatory response or it may indicate the deleterious synergic effect of these cytokines on tissues. In the study by Schots,^22^ the multivariate analysis showed that the increase of these cytokines, especially IL-8, was associated with a fatal result in HSCT. There is also a study that relates IL-8 to the adult respiratory distress syndrome.^43^

The study by Prat et al. evaluated several biomarkers (PCT, CRP, neopterin, IL-6 and IL-8) in 61 adults (with 41 submitted to HSCT, and the other ones only under chemotherapy). IL-6 and IL-8 did not prove useful to prevent bloodstream infection before chemotherapy, on the first day of neutropenia nor subsequent days.^34^

## Studies of Inflammation Marker and Poor Prognosis (Death) Related to HSCT

An ideal death marker in the neutropenic population after HSCT is the one that indicates, at the onset of fever, or at most up to 24 hours after, the patients under potential risk of death transplant-related. There are few studies in literature to help the prediction of the prognosis of patients submitted to HSCT and the existing studies evaluate a small number of subjects. The following table shows the main studies that evaluated death predictors after HSCT (Table 2). The studies published about the prognosis in patients after HSCT are divided into allogeneic or autologous. Despite the advance in supporting treatment, the allogeneic HSCT still presents high toxicity, with mortality related to transplantation between 10–50% due to major complications during the first months after transplantation (infections, VOD, GVHD and pneumonitis).

In a prospective study of 296 patients submitted to HSCT at our university hospital, IL-6, PCT and CRP were evaluated on the day of confirmed neutropenia, on the day of the febrile event, 24 and 72 hours after its onset, and 48 hours or 5 days, in case the fever, persisted.^46^ The patients were evaluated after the discharge or death within 30 days from the HSCT. Only CRP ≥120 mg/L was independently associated with death. The other risk factors associated with death in the multivariate analysis were: type of transplantation (allogeneic and unrelated), bloodstream infection by Gram-negative, LDH≥390 IU/L and urea ≥25 mg/dL. For allogeneic patients, only CRP≥120mg/L and bloodstream infection by Gram-negative were risk factors for death. No independent risk factor was evidenced for the subgroup of autologous patients. We could not demonstrate the association between IL-6 and PCT and death. Other authors demonstrated similar results, however, in studies with a smaller number of cases and or without multivariate analyzes or testing only one or two biomarkers.^21^,^22^,^35^,^37^ Artz et al.^42^ utilized multivariate analysis, however, with a smaller number of patients (n = 112) and only analyzed two markers (CRP and IL-6) in allogeneic HSCT. In their study, the median of CRP was 18 mg/L, and of IL-6 was 78 pg/mL, showing that high levels of CRP, but not of IL-6, before the conditioning for HSCT, were independent predictors of death related to allogeneic transplantation. The study of Remberger et al. (2010),^21^ despite including a large number patients,299, was retrospective, without multivariate analysis and, likewise the two previous ones, evaluated only CRP in allogeneic HSCT before the conditioning. The authors showed that mortality related to transplantation was lower in patients with non-myeloablative regimens with high CRP (67 vs 43%, p = 0.005, and 16 vs 30%, p = 0.036). Finally, the study by Ortega et al. (2004)^35^ analyzed only CRP, did not utilize multivariate analysis and the number of 100 (smaller than that of the present study), in autologous and allogeneic patients, and showed that CRP on the 5th day of fever ≥ 16 mg/dL was associated with death, due to infectious causes with a sensitivity of 100%, a specificity of 87%, PPV and NPV of 30 and 100%, respectively.

Schots et al. (2002)^22^ monitored the levels of CRP and other variables in 96 consecutive adults submitted to allogeneic HSCT, dosing from day 0 to +5. Only the high levels of CRP (> 50 mg/L) (p < 0.001) and type of allogeneic donor (p = 0.02) were the independent factors for mortality related to transplantation. The retrospective study of Remberger et al. (2010)^21^, evaluated the clinical impact of CRP in allogeneic HSCT patients, before the conditioning (n = 205) and after the not-myeloablative and myeloablative conditioning regimens (n = 299). There was a significant correlation between CRP levels in both groups with overall survival and mortality related to transplantation increased in individuals who had received non-myeloablative conditioning. Early death by infection (within 100 days) resulted in 25 and 8.7% of patients with high CRP with and without documented infection, respectively (p < 0.001 and p = 0.04), compared to patients with normal CRP, where early death by infection occurred in 2.3% of patients. Remberger et al. (2010)^21^ concluded that CRP may be utilized as a prognostic factor because it enables the identification of infection in earlier stages, as also observed in our results. In the studies by Pihusch et al., the serum levels of CRP (5.2 mg/dL vs 7.8 mg/dL, p < 0.001), IL-6 (147 ng/mL vs 494 ng/mL; p < 0.001) and PCT (2.1 ng/mL vs 4.0 ng/mL; p = 0.003) were high until week +2 in the group of patients who died of complications related to transplantation, compared to the group that survived.

## Perspectives

The treatment of febrile neutropenic patients after HSCT requires proper and early diagnosis. Thus, biomarkers can be very useful to control the antibiotic use in this setting. Sensitive and specific lab markers in differentiating the infectious and non-infectious origin of the fever may help the proper use of antibiotics, thus leading to cost reduction. Until now, however, the role of biomarkers as tools in the conduction of the febrile neutropenia is questionable. The latest guide from the Infectious Disease Society of America (IDSA) does not recommend the use of those biomarkers.^49^ Therapeutic decisions are still based on the combination of anamnesis, physical examination and microbiological tests. An ideal marker is the one that can be easily obtained from clinical specimens, before the development of severe sepsis. It should not be influenced by cytopenias or inflammatory states associated with the underlying disease. Besides, it is always hard to correlate laboratory findings and clinical events, mainly in such peculiar population. An ideal marker should be easily detected in blood samples obtained before the development of more severe sepsis and should not be influenced by cytopenias, drugs, or by the inflammatory reaction to the underlying disease. IL-8 is also a marker studied quite often but does not have these characteristics.

## Figures and Tables

**Table 1:** The most important studies with adults patients submitted to HSCT comparing use of biomarkers to predict infections.

Author	Biomarkers	Patients	Study Design	Results	Comments
Ortega et al. (2004)	PCT	- 77 adults - Allogeneic and autologous	- 3 times/week since the 1^st^ day of fever until the resolution of it - 4 Groups: (i) documented infection; (ii) clinical infection; (iii) fever of non-infectious origin; (iv) FUO	- 1^st^ day of fever: average (±SD) PCT 0.3 ng/L Group iv FUO - 0.5 ng/L in documented infection (i); 0.2 ng/mL in clinical infection (iii) and 1.7 in fever of non-infectious origin (iv) (p = NS) - Confirmed cases of invasive aspergilosis PCT very high 10.1 ng/mL in comparison with the other groups p = 0.027	- PCT on the 1^st^ day of fever did not enable differential diagnosis of fever. - If fever persisted for more than five days, values of PCT ≥ 3 ng/mL had more sensitivity and specificity for the diagnosis of invasive aspergilosis.
Pihusch et al. (2006)	PCT, IL-6 and CRP	- 350 - > 12 years - Allogeneic Related *Full match*	- Conditioning before and once a week for eight weeks after HSCT	- PCT (0.8 ng/dL vs. 5.7 ng/dL; P< 0.001) - IL-6 (9.3 ng/mL vs. 1.138 ng/mL; p < 0.001) - CRP (4.4 mg/dL vs. 12.8 mg/dL; p,0,0.001) in infectious complications	- CRP, IL-6 and PCT are useful markers for non-infectious complications - PCT can differentiate infection from aGVHD -PCT did not reduce with corticoid in aGVHD (2.3 ng/dL vs. 2.0 ng/dL; NS)
Prat et al. (2008)	PCT, CRP, neopterin, IL-6 e IL-8	- 61 adults (41 HSCT, others CHT)	- CHT before, first day neutropenia and every 24 hours until 6 days - Febrile episodes divided into two groups: Group 1 (confirmed micro infection) and Group 2 (FUO)	- Higher levels of PCT and CRP at the onset of fever compared to diagnosis (p = 0.014 and p < 0.001) - Neopterin without difference (p = 0.290) - Globally, without differences between the two groups for PCT (p = 0.724), CRP (p = 0.283), neopterin (p = 0.876), IL-6 (p = 0.169) and IL-8 (p = 0.150) - Best cut-off value for PCT to predict BSI by Gram-negative was 1 ng/ML	- IL-6 and IL-8 were not useful to predict BSI - CRP was more sensitive than PCT, but the specificity was low - Neopterin was not useful because it increases with the use of G-CSF - PCT has a high NPV for BSI by Gram-negative - CRP was better to predict BSI (both Gram-positive and Gram-negative), but with low specificity, with cut-off value of 135 μg/mL.
Azarpira et al. (2009)	PCT and CRP	- 35 adults - Allogeneic and autologous	- D 0 before HSCT and one and three weeks after	- CRP was higher in GVHD vs. the patients with GVHD (20 ± 2.5 mg/l vs. 10.12 ± 5.2 mg/L – p = 0.06) - PCT increased in patients with bacterial sepsis (e.g.: > 10 ng/mL)	- PCT and CRP are biomarkers of post-Tx complications, such as GVHD and infections and PCT may differentiate GVHD infections

Tx = transplantation; D= day; mPCT = mean PCT; pCRP = mean CRP; AUC = area under curve; NS = non-significant; FN = febrile neutropenia; ATG = anti-thymocyte globulin; CHT = chemotherapy; FUO = fever of unknown origin; BSI = bloodstream infection; G-CSF = granulocyte colony stimulating factor; NPV = negative predictive value; GVHD = graft versus host disease; aGVHD = acute graft versus host disease; ESR = erythrocyte sedimentation rate; NS = non-significant; Ang-1 = serum angiopoietin 1; Ang-2 serum angiopoietin 2; CI = confidence interval; SD = standard deviation.

**Table 2:** The most important studies with adults patients submitted to HSCT comparing the use of biomarkers as predictors of death.

Author	Biomarkers	Patients	Study Design	Results	Comments related to prognosis
Schots et al. (1998)	CRP	N=66->15 years Allogeneic Related or unrelated	Day 0 or day 1 after HSCT and every 24 or 48 hours until death (at least 3 weeks after Tx)	CRP levels >200 mg/mL in 80% of the cases with severe complications and levels >300 mg/mL associated to death in 31% (p<0,001)	High levels of CRP after allogeneic HSCT are associated to death.
Schots et at. (2002)	CRP	N=96 consecutive adults (age 15–50) allogeneic related or unrelated	Every 2 days or daily during fever (from the day 0 of HSCT until marrow recovery-discharge or death)	Levels of CRP between days 5–10 and probability of mortality related to HSCT: < 100 mg/L 1% in related full match > 100 mg/L 36.5% in full match	High levels of CRP on the first 5–10 days after HSCT were associated to death.
Schots et at. (2003)	IL-6, IL-8 e TNF-α	N=84 adults (>15 years) Allogeneic Related or unrelated	Collections every 2 days or daily in the febrile period from D0 of HSCT until marrow recovery and discharge or death.	Mortality related to HSCT focused on the first 10 days after HSCT: medians (pg/mL): 69 (8–2934) (IL-6), 13 (2–474) (IL-8) and 10 (3–75) (TNF).	Multivariate analysis showed that the increase of those cytokines, especially IL-8, was associated to death.
Ortega et al. (2004)	CRP	N=100 consecutive adults Allogeneic and autologous	CRP analyses every 48 h from admission until the resolution of the febrile episode.	CRP on the 5^th^ day of fever ≥ than 16 mg/dL was associated to death due to infectious causes with sensitivity of 100%, specificity of 87%, PPV and NPV of 30% to 100%, respectively.	CRP above 16 mg/dL on the 5^th^ day of fever was strongly predictive of death associated with infection.
Artz et al. (2008)	CRP e IL-6	N=112 allogeneic adults Related or unrelated	IL-6 and CRP cryopreserved for 2 weeks or less before HSCT conditioning	The median of CRP was a little high (18 mg/L). Likewise, the median of IL-6 levels was also remarkably high in this study (78 pg/mL).	In multivariate analysis CRP was predictive of death not due to recurrence (p= 0.04). The level of IL-6 did not have significant relationship with death.
Remberger et al. (2010)	CRP	N=299 adults Allogeneic either related or not	CRP dosed before conditioning (evaluated retrospectively)	Overall survival and mortality related to the HSCT were worse in patients with non-myeloablative regimes with high CRP (67 vs 43%, p= 0.005, and 16 vs 30%, p= 0.036.	Patients who had already started the conditioning with infection and high CRP (n=16) had the worst end result.
Massaro etal 2014	CRP. IL-6, PCT	N=296 adults allogeneic and autologous	IL-6, PCT and CRP) were assessed on the day afebrile neutropenia, in the febrile event, 24 and 72 h after fever onset and 48 h or 5 days if fever per-sisted.	Only CRP> 120 mg/L was independently associated with death.	For allogeneic patients only CRP >120 mg/L and BSI due to Gram-negative were risk factors for death; however, CRP did not remain in the model when urea >25 mg/L was included.

Tx = transplantation; FN = febrile neutropenia; ATG = anti-thymocyte globulin; CHT = chemotherapy; FUO = fever of unknown origin; BSI = bloodstream infection; NPV = negative predictive value; GVHD = graft versus host disease; aGVHD = acute graft versus host disease; ESR = erythrocyte sedimentation rate; NS = non-significant; CI = confidence interval; SD = standard deviation.
